# Remote Intracranial Hemorrhage after Cranio-Spinal Surgery. Report of Two Cases

**DOI:** 10.5334/jbsr.2259

**Published:** 2020-11-30

**Authors:** Steffy Larroze, Valentina Lolli, Niloufar Sadeghi

**Affiliations:** 1 Erasme Hospital, BE

**Keywords:** Remote cerebellar hemorrhage, intracranial hemorrhage, dural tear, CT, MRI

## Abstract

Post-operative intracranial bleeding at a distance from the surgical site is a rare complication of both cranial and spinal surgeries and is referred to as remote intracranial hemorrhage (RIH). Bleeding typically occurs in the cerebellum. Simultaneous hemorrhages in different cranial compartments have been rarely observed. We herein report two cases of RIH, which showed different imaging patterns and clinical signs and symptoms. RIH are typically self-limiting and do not usually require treatment. Physicians must be aware of this benign entity which needs not be misdiagnosed with other conditions.

**Teaching point:** Remote intracranial hemorrhage is a rare but worthy of note complication of cranio-spinal surgery.

## Introduction

Post-operative intracranial bleeding occurring at a distance from the surgical site is a rare complication of both supra- and infra-tentorial craniotomies as well as spinal surgeries and is referred to as remote intracranial hemorrhage (RIH). The most common location is the cerebellum [1,2]. Combination of intra- and extra-parenchymal hemorrhages in both supra- and infra-tentorial compartments is exceedingly rare as only a few cases have been reported [3,4,5,6,7,8,9]. We herein present two cases of RIH, which showed different imaging patterns and clinical signs and symptoms.

## Case 1

**Figure 1:**
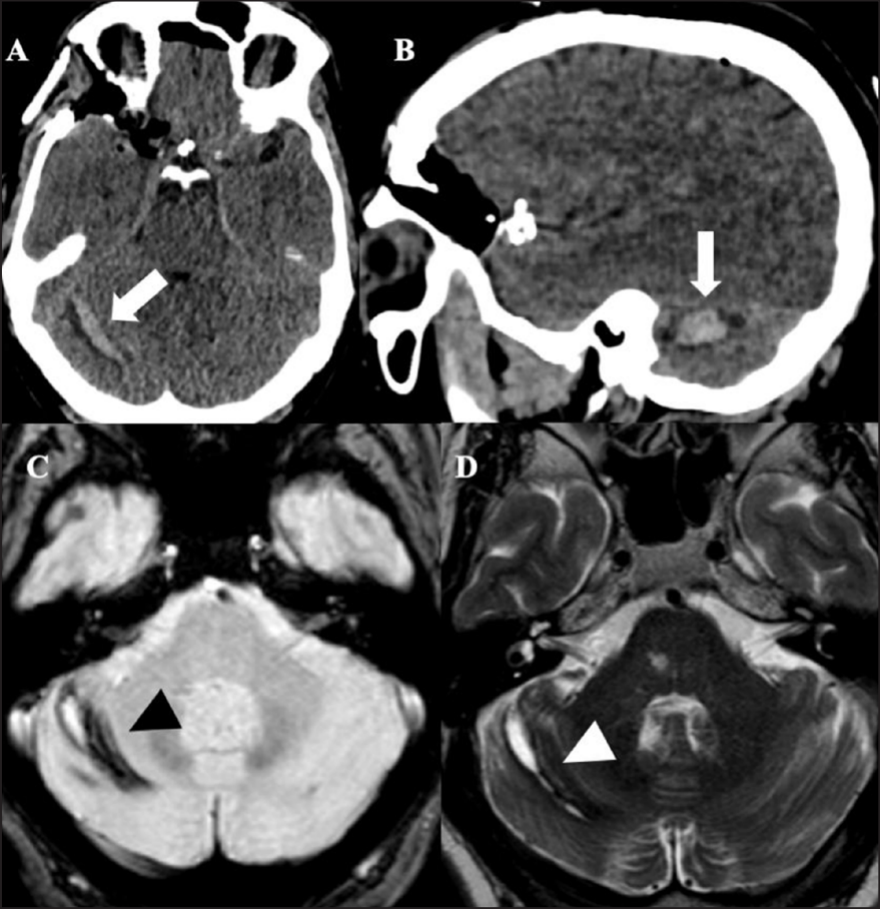
**Patient 1. (A, B)** Axial and sagittal CT images show a right cerebellar hemorrhage (*arrow*) exhibiting multiple stripes referred to as the ‘*zebra sign*’. **(C)** Axial T2 gradient-echo and **(D)** axial T2 weighted-images confirm hemosiderin deposits in the right cerebellar hemisphere (*arrowheads*).

A 54-year-old male underwent a right pterional craniotomy for clipping of two unruptured aneurysms. A right cerebellar hemorrhage was noted on post-operative brain CT and confirmed on MRI (Figure 1).

## Case 2

**Figure 2:**
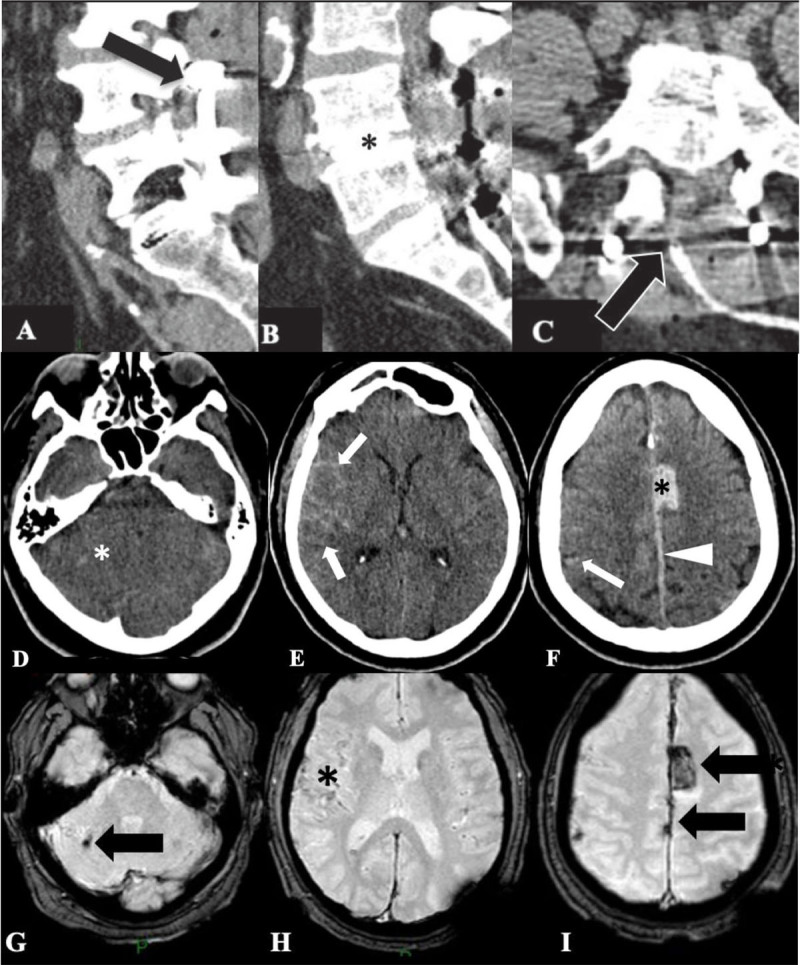
**Patient 2. (A, B)** Sagittal lumbar CT images show the L4-L5-S1 posterior arthrodesis (*arrow*) and implementation of L4-L5 disc prosthesis (*); **(C)** Axial CT image at the L5-S1 level shows a catheter at the site of the laminectomy (*arrowhead*). **(D–F)**: Axial brain CT images show a tiny right cerebellar hematoma (D, *), diffuse subarachnoid hemorrhage (E, F, *arrows*), a left interhemispheric subdural hematoma (F, *arrowhead*) and a parasagittal left superior frontal intraparenchymal hematoma (F, *).

A 60-year-old male underwent decompressive lumbar surgery. Post-operatively, an excessive volume of drainage fluid was collected by the catheter that had been left in place (Figure 2A, B, C), suggesting an undetected dural tear. The patient developed generalized seizures. Brain CT demonstrated hemorrhages in different intracranial compartments (Figure 2D, E, F), with no underlying vascular abnormality. Findings were confirmed on MRI (Figure 2G, H, I).

Both patients were treated conservatively. One month afterwards, they were in good clinical condition and brain imaging showed no residual hemorrhage.

## Discussion

Case 1 is a classic presentation of remote cerebellar hemorrhage complicating a supratentorial craniotomy. CT demonstrates a pathognomonic imaging pattern of alternating hyperdense and hypodense stripes following the cerebellar folia, referred to as the zebra-sign because of its resemblance to the animal’s black and white coat [10]. Patient 2 conversely associates subdural, subarachnoid, and parenchymal hemorrhages, which have been rarely observed simultaneously [3,4,5,6,7,8,9].

The exact incidence of RIH associated with spinal surgery is unknown because brain CT is not systematically performed post-operatively, but it seems low, varying from 0.08 to 0.4% [8,9]. The pathophysiological mechanism underlying RIH is not yet fully elucidated but a massive loss of CSF during and/or after the procedure seems to be involved, leading to intracranial hypotension and tearing with subsequent rupture of bridging veins [6,8,11]. Indeed, dural tears are common, with a 1–17% incidence and may result in RIH in all brain compartments [7,9]. The hypothesis of a venous etiology is supported by the frequently observed bilateral distribution of hemorrhage, in contrast to arterial bleeds which are typically unilateral [11].

RIH are typically self-limiting, and usually do not require any specific treatment. Thus, this benign entity needs not be misdiagnosed with other more severe etiologies, such as hypertensive hemorrhages or ruptured vascular malformations.

## Conclusion

Physicians must be alert to the possible development of RIH post-operatively and aware of their benign course.
